# Treatment satisfaction in type 2 diabetes patients taking empagliflozin compared with patients taking glimepiride

**DOI:** 10.1007/s11136-015-1140-2

**Published:** 2015-09-30

**Authors:** Costel Chirila, Qingyao Zheng, Eric Davenport, Dagmar Kaschinski, Egon Pfarr, Thomas Hach, Roberto Palencia

**Affiliations:** RTI Health Solutions, 200 Park Offices Drive, Research Triangle Park, NC 27709 USA; Boehringer Ingelheim GmbH, Binger Str. 173, 55216 Ingelheim am Rhein, Germany; Boehringer Ingelheim Pharma GmbH & Co. KG, Binger Str. 173, 55216 Ingelheim am Rhein, Germany

**Keywords:** Type 2 diabetes mellitus, Diabetes Treatment Satisfaction Questionnaire, Empagliflozin, Glimepiride

## Abstract

**Purpose:**

This exploratory analysis assessed and compared patients’ treatment satisfaction with empagliflozin plus metformin versus glimepiride plus metformin, using data obtained from the Diabetes Treatment Satisfaction Questionnaire, status version (DTSQs) collected in a randomized, double-blind, double-dummy clinical trial.

**Methods:**

Observed values for DTSQs scale score and each of its eight items were summarized by visit and treatment arm. Changes from baseline in these scores were analyzed using linear mixed models for repeated measures.

**Results:**

The baseline scale score and item scores were comparable between empagliflozin plus metformin (*n* = 765) and glimepiride plus metformin (*n* = 780). Compared with baseline, patients reported significant treatment satisfaction increases and significant decreases in perceived hyperglycemia with both treatments at all visits. Also, compared with baseline, a significant increase in perceived frequency of hypoglycemia was observed in the glimepiride treatment group at all visits. No statistically significant treatment difference was observed in DTSQs scale score and its items at week 104. The difference between the treatment groups was significant and in favor of empagliflozin from week 28 onward for perceived frequency of hyperglycemia (*P* ≤ 0.006) and perceived frequency of hypoglycemia (*P* ≤ 0.011).

**Conclusions:**

Despite positive trends in favor of empagliflozin, there was no significant difference in DTSQs scale score between empagliflozin and glimepiride at 104 weeks. However, when compared with glimepiride, empagliflozin demonstrated significantly lower perceived frequency of hyperglycemia and hypoglycemia at all visits from week 28 onward. This finding is consistent with the clinical results reported for the EMPA-REG H2H-SU trial.

## Introduction

Diabetes is a disease with a large and increasing societal cost due to the number of people affected worldwide and its associated complications, such as increased risk of cardiovascular disease, neuropathy, and eye complications. In 2013, 382 million patients worldwide were estimated to have diabetes, of which 46 % (175 million) were undiagnosed. The number of patients living with diabetes is estimated to increase by 55 % (592 million) by 2035 [1]. The majority of patients with diabetes have type 2 diabetes mellitus (T2DM), approximately 85–95 % in high-income countries and even more in low- and middle-income countries [1]. T2DM is characterized by insulin resistance and a progressive decrease in the ability of the beta cells in the pancreas to produce sufficient amounts of insulin to control blood glucose.

Quality of life and patient-reported outcome (PRO) measures are collected in clinical trials because they offer information from the patient perspective, which is beyond the usual efficacy and safety profiles of a drug. PROs measuring treatment satisfaction have an important place in diabetes management because treatment satisfaction is important to patients and because better treatment satisfaction may have a positive impact on treatment adherence and self-management behavior [2]. The Diabetes Treatment Satisfaction Questionnaire, status version (DTSQs) is a PRO instrument which, according to the World Health Organization and the International Diabetes Federation, is “useful in assessing outcomes of diabetes” [3]. The DTSQs has been used in several studies to measure treatment satisfaction and has shown sensitivity to changes in treatments [4].

When T2DM is diagnosed, clinical guidelines recommend starting treatment with changes in lifestyle, such as diet and exercise; however, as the disease progresses, there remains a need for antidiabetic drugs. According to the American Diabetes Association and the European Association for the Study of Diabetes, first-line treatment for the management of hyperglycemia in patients with T2DM consists of changes in lifestyle plus metformin; second-line treatment consists of changes in lifestyle plus metformin and sulfonylurea [5]. Other new classes of antidiabetic agents, such as sodium-dependent glucose cotransporter 2 (SGLT-2) inhibitors, have also been introduced to the market; these treatments are now being considered to determine treatment algorithms for hyperglycemia in patients with T2DM.

The SGLT-2 is expressed in the renal proximal tubules and accounts for 90 % of the total renal glucose reabsorption in healthy individuals [6, 7]. Empagliflozin is an oral antidiabetic drug that selectively inhibits the SGLT-2 and increases urinary glucose excretion by blocking glucose reabsorption by the kidney. Treatment with empagliflozin in phase three clinical trials resulted in clinically meaningful reductions in glycated hemoglobin (HbA1c), systolic blood pressure, and body weight. Empagliflozin also demonstrated good overall safety and tolerability in patients with T2DM and showed a low risk of hypoglycemia [8–11]. EMPA-REG H2H-SU was one of the phase three clinical trials in which patients with T2DM who had insufficient glycemic control despite taking metformin were randomized to either empagliflozin or glimepiride as add-on treatment to metformin. Glimepiride is widely used and available at a reasonable price in many countries for treatment of type 2 diabetes as monotherapy or add-on therapy to metformin when diet and physical exercise and weight reduction alone are not adequate [12, 13].

The objective of this exploratory analysis was to assess and compare patients’ treatment satisfaction with empagliflozin plus metformin versus glimepiride plus metformin using data obtained from the DTSQs collected in the EMPA-REG H2H-SU trial.

## Methods

Data for this study were obtained from the EMPA-REG H2H-SU clinical trial. This trial is described in detail in Ridderståle et al. [11] and is briefly summarized here. After a two-week open-label, placebo run-in period, 1549 patients with T2DM and insufficient glycemic control (HbA1c from 7.0 to 10 % and body mass index ≤45 kg/m^2^ at screening) were randomly assigned to receive for 104 weeks either empagliflozin 25 mg orally once a day (*n* = 769, of which 765 received treatment) or glimepiride 1–4 mg orally once a day (*n* = 780) as an add-on therapy to their current treatment of immediate-release metformin [11]. Randomization was performed via an interactive voice response system in 23 countries (Argentina, Austria, Canada, Colombia, Czech Republic, Finland, Hong Kong, India, Italy, Malaysia, Mexico, Netherlands, Norway, Philippines, Portugal, South Africa, Spain, Sweden, Switzerland, Taiwan, Thailand, United Kingdom, USA), and study medication was dispensed in a double-blind, double-dummy manner. The metformin dose (unchanged for 12 weeks prior to randomization) was ≥1500 mg per day, or the maximum dose tolerated, or the maximum dose according to the local label. The starting dose of glimepiride was 1 mg/day and then was up-titrated 1 mg/day every 4 weeks during the first 12 weeks of the treatment period up to the maximum of 4 mg/day if fasting home blood glucose monitoring values were >110 mg/dL. Up-titration could be withheld during the first 12 weeks, or down-titration could occur after the first 12 weeks if the patient was at increased risk of hypoglycemia. All patients enrolled in the study provided informed consent. The trial was conducted according to the principles of the Declaration of Helsinki and the International Conference on Harmonisation’s Harmonised Tripartite Guideline for Good Clinical Practice [11].

The main objective of the trial was to investigate the efficacy, safety, and tolerability of empagliflozin 25 mg compared with glimepiride 1–4 mg. The primary endpoint was the change from baseline in HbA1c after 104 weeks of treatment. Key secondary endpoints were occurrence of confirmed hypoglycemic adverse events (plasma glucose ≤3.9 mmol/L or requiring assistance) and change from baseline in body weight and systolic and diastolic blood pressure after 104 weeks of treatment. The PRO measures that were included in the trial were the EuroQol 5 Dimensions health questionnaire (3 levels) and the DTSQs, and both were administered at baseline and weeks 8, 28, 52, 74, and 104. Healthcare resource utilization was also collected throughout the trial.

The DTSQs (available from www.healthpsychologyresearch.com), which is the status version of the questionnaire, has a total of eight items: six items assessing treatment satisfaction (i.e., overall treatment satisfaction, treatment convenience, treatment flexibility, satisfaction with understanding of diabetes, willingness to continue present treatment, and willingness to recommend present treatment to others) and two items assessing perceived frequency of unacceptably high blood glucose levels (hyperglycemia) and unacceptably low blood glucose levels (hypoglycemia) [14, 15]. Patient responses to each DTSQs treatment satisfaction item are reported on a 7-point Likert scale, with 6 being very satisfied, very convenient, and very flexible and 0 being very dissatisfied, etc. The DTSQs scale score is calculated by summing the six individual treatment satisfaction item scores; scale scores can range between 0 and 36, with higher scores indicating more satisfaction with treatment. The DTSQs scale score was set to missing if any of the six individual items were missing. The questions assessing hyperglycemia and hypoglycemia are stand-alone items and are treated separately from treatment satisfaction. These two items also are reported on a 7-point Likert scale between 6 and 0; for these two questions, lower scores indicate fewer episodes of hyperglycemia or hypoglycemia.

The analysis population for the DTSQs scale score, perceived hyperglycemia, and perceived hypoglycemia consisted of all patients in the full analysis set (i.e., all randomized patients treated with at least one dose of the study drug and with a baseline HbA1c measurement) with a baseline and at least one postbaseline DTSQs measurement (i.e., DTSQs scale score, perceived hyperglycemia, perceived hypoglycemia).

The number and percentage of patients who completed DTSQs assessments were reported for each scheduled visit. Summary tables were created based on the observed values by visit and treatment arm for DTSQs scale score and each of the eight items assessed in the questionnaire. Changes from baseline in DTSQs scale score and each of the eight item scores were analyzed using linear mixed models for repeated measures across postbaseline visits. The models included treatment, visit, and interaction between treatment and visit as fixed effects, regardless of their significance. A random intercept for patients was also programmed into the models to account for within-patient correlations. In addition, a pool of potential adjustment covariates was reviewed for inclusion into each of the models using a backward-selection process.

Continuous variables included baseline values of DTSQs, age, body mass index, HbA1c, and systolic and diastolic blood pressure; and categorical variables included baseline values of estimated glomerular filtration rate (eGFR), time since diagnosis, sex, race, country, prior cardiovascular event, and cardiovascular risk predictors, defined as yes/no, where “yes” meant the occurrence of at least one of the following events: blood pressure (systolic/diastolic) >140/90 mmHg, or HbA1C level at baseline ≥8.5, or eGFR at baseline ≤59, or a prior cardiovascular event occurred. Due to the exploratory nature of this analysis, variables with a *P* value ≤0.10, rather than the usual 0.05, were selected for the final adjusted models through the backward selection.

The same models were fitted for DTSQs scale score and its individual items to ensure consistency and comparability; different models were fitted for the stand-alone perceived hyperglycemia and perceived hypoglycemia items. Adjusted means by treatment and differences in adjusted means were estimated at each visit, but the primary visit for the analyzed endpoints was 104 weeks. Due to the exploratory nature of the analysis, no adjustment for multiplicity was performed. A *P* value of 0.05 was used to determine statistical significance.

## Results

The EMPA-REG H2H-SU clinical trial’s results were described in detail in Ridderståle et al. [11] and are briefly summarized here. When compared to patients taking glimepiride added to metformin, patients taking empagliflozin added to metformin showed a sustained reduction in HbA1c, significant at week 104 (adjusted mean difference of −0.11 %, *P* = 0.0153 for superiority in favor of empagliflozin; 95 % confidence interval [CI] −0.19 to −0.02 %). A sustained and significant difference in body weight and blood pressure, in favor of empagliflozin, was also noted in all visits. Furthermore, significantly fewer patients taking empagliflozin had confirmed hypoglycemic adverse events than patients taking glimepiride within 104 weeks (relative risk ratio adjusted for baseline HbA1c (<8.5 vs. ≥8.5 %) was 0.102 (95 % CI, 0.065–0.162). Based on the adverse events with a frequency of at least 10 % in each treatment group, a higher percentage of the following adverse events was observed in the glimepiride treatment group than in the empagliflozin treatment group: hyperglycemia (22 vs. 14 %) and hypertension (10 vs. 5 %). A higher percentage of one or more serious adverse events (16 vs. 11 %) and of events consistent with genital infections (12 vs. 2 %) was observed in empagliflozin than in glimepiride. The percentage of events consistent with urinary tract infection was similar between the treatment groups (13 % in glimepiride vs. 14 % in empagliflozin).

The demographic and baseline characteristics were comparable between the treatment groups (Table 1).

**Table 1:** Demographic and baseline characteristics for full analysis set population

Characteristic	Empagliflozin 25 mg (*N* = 765)	Glimepiride 1–4 mg (*N* = 780)	Overall (*N* = 1545)
Sex [*n* (%)]			
Male	432 (56.5)	421 (54.0)	853 (55.2)
Female	333 (43.5)	359 (46.0)	692 (44.8)
Race [*n* (%)]			
Asian	254 (33.2)	253 (32.4)	507 (32.8)
Black/African American	12 (1.6)	8 (1.0)	20 (1.3)
Other (i.e., American Indian/Alaska Native, Hawaiian/Pacific Islander)	1 (0.1)	0	1 (0.1)
White	498 (65.1)	519 (66.5)	1017 (65.8)
Country [*n* (%)]			
United States	43 (5.6)	51 (6.5)	94 (6.1)
Other	722 (94.4)	729 (93.5)	1451 (93.9)
Age group [years, *n* (%)]			
<50	197 (25.8)	212 (27.2)	409 (26.5)
50–65	420 (54.9)	434 (55.6)	854 (55.3)
>65	148 (19.3)	134 (17.2)	282 (18.2)
Age [years, mean (SD)]	56.20 (10.30)	55.67 (10.44)	55.93 (10.37)
Baseline BMI [*n* (%)]			
<25	131 (17.1)	112 (14.4)	243 (15.7)
25–30	289 (37.8)	309 (39.6)	598 (38.7)
>30	345 (45.1)	359 (46.0)	704 (45.6)
Baseline BMI [kg/m^2^, mean (SD)]	29.95 (5.28)	30.27 (5.30)	30.11 (5.29)
Baseline HbA1c [*n* (%)]			
<7.5	250 (32.7)	281 (36.0)	531 (34.4)
7.5 to <8.5	334 (43.7)	308 (39.5)	642 (41.5)
8.5 to <9.5	138 (18.0)	146 (18.7)	284 (18.4)
≥9.5	43 (5.6)	45 (5.8)	88 (5.7)
Baseline HbA1c [%, mean (SD)]	7.92 (0.81)	7.92 (0.86)	7.92 (0.84)
Baseline systolic blood pressure, seated [mmHg, mean (SD)]	133.42 (15.92)	133.54 (15.98)	133.48 (15.95)
Baseline diastolic blood pressure, seated [mmHg, mean (SD)]	79.54 (9.59)	79.38 (9.24)	79.46 (9.41)
Blood pressure [systolic/diastolic, mmHg, mean (SD)]			
<120/<80	346 (45.2)	350 (44.9)	696 (45.1)
120–140/80–90	167 (21.8)	169 (21.7)	336 (21.7)
>140/>90	252 (33.0)	261 (33.5)	513 (33.2)
Baseline eGFR [*n* (%)]			
≥90	313 (40.9)	318 (40.8)	631 (40.8)
60 to <90	439 (57.4)	440 (56.4)	879 (56.9)
30 to <60	13 (1.7)	22 (2.8)	35 (2.3)
Baseline eGFR (MDRD) [mL/min/1.73 m^2^, mean (SD)]	87.94 (16.82)	88.11 (17.85)	88.02 (17.34)
Time since diagnosis of T2DM [years, *n* (%)]			
≤1	79 (10.3)	93 (11.9)	172 (11.1)
>1–5	341 (44.6)	336 (43.1)	677 (43.8)
>5–10	214 (28.0)	211 (27.1)	425 (27.5)
>10	131 (17.1)	140 (17.9)	271 (17.6)
Prior cardiovascular events [*n* (%)]			
Yes	152 (19.9)	155 (19.9)	307 (19.9)
No	613 (80.1)	625 (80.1)	1238 (80.1)
Cardiovascular risk predictor [*n* (%)]^a^			
Yes	442 (57.8)	469 (60.1)	911 (59.0)
No	323 (42.2)	311 (39.9)	634 (41.0)

*BMI* body mass index, *eGFR* estimated glomerular filtration rate, *HbA1c* glycated hemoglobin, *MDRD* modification of diet in renal disease, *SD* standard deviation, *T2DM* type 2 diabetes mellitus

^a^Defined as yes/no, where “yes” meant the occurrence of at least one of the following events: blood pressure (systolic/diastolic) >140/90 mmHg, or HbA1C level at baseline ≥8.5, or eGFR at baseline ≤59, or a prior cardiovascular event occurred

The completion rate for the DTSQs instrument was high, and the rate was similar between the treatment arms; completion rates were 92 % or greater up to 52 weeks and almost 92 % after 52 weeks (Table 2).

**Table 2:** DTSQs completion rates

Time	Empagliflozin 25 mg	Glimepiride 1–4 mg	Overall
All randomized analysis set (*N*)	765	780	1545
DTSQs analysis set [*n* (%)]^a^	718 (94 %)	742 (95 %)	1460 (94 %)
Completed DTSQs [*n* (%)]			
Baseline	718 (100 %)	742 (100 %)	1460 (100 %)
Week 8	713 (99 %)	736 (99 %)	1449 (99 %)
Week 28	690 (96 %)	705 (95 %)	1395 (96 %)
Week 52	666 (93 %)	685 (92 %)	1351 (93 %)
Week 78	649 (90 %)	659 (89 %)	1308 (90 %)
Week 104	621 (86 %)	634 (85 %)	1255 (86 %)

*DTSQs* Diabetes Treatment Satisfaction Questionnaire, status version

^a^DTSQs analysis set is defined as all patients having a baseline glycated hemoglobin measurement, a baseline DTSQs assessment, and at least one postbaseline DTSQs assessment

**Fig. 1:**
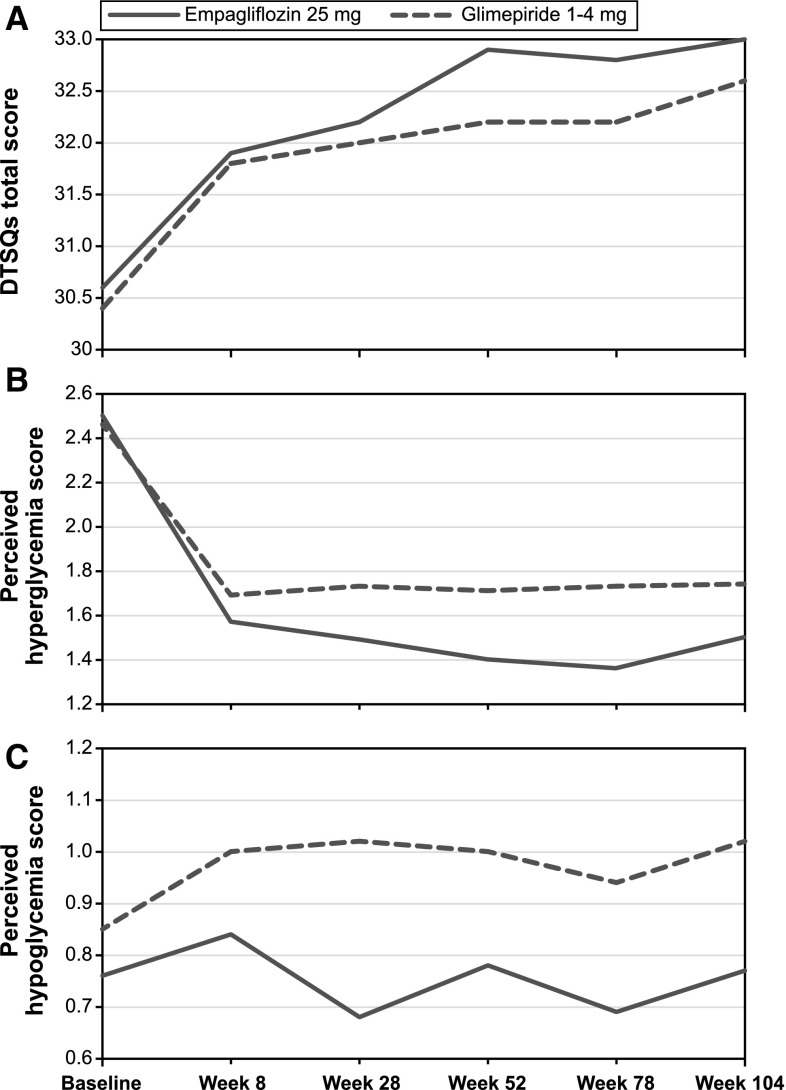
Unadjusted mean scores by time: empagliflozin 25 mg versus glimepiride 1–4 mg. *DTSQs* diabetes treatment satisfaction questionnaire, status version

The baseline DTSQs scale score was comparable between the treatment arms, with an unadjusted mean of 30 (out of a maximum of 36), indicating relatively high satisfaction. The mean DTSQs scale score increased slowly but steadily over time in both treatment arms and was slightly larger for the empagliflozin arm than for the glimepiride arm from week 52 onwards (Fig. 1a). The mean perceived hyperglycemia score, which was low and similar at baseline (mean, 2.5), decreased sharply for both arms at week 8 and then remained almost constant for glimepiride, while scores decreased further for empagliflozin (Fig. 1b). The mean baseline score for perceived hypoglycemia was 0.76 for empagliflozin and 0.85 for glimepiride. The scores fluctuated around the baseline mean for patients taking empagliflozin, whereas scores increased in glimepiride patients (Fig. 1c).

Table 3 presents adjusted mean scores at each postbaseline visit and the corresponding treatment differences in changes from baseline in DTSQs scale score and its individual items. The covariates selected for the final adjusted model for DTSQs total score and its individual items were baseline DTSQs scale score, diastolic blood pressure, and race. Within each treatment arm, significant increases from baseline in treatment satisfaction were observed for DTSQs scale score and its individual items at all visits. Between the two treatment arms, no significant differences in the adjusted mean change from baseline were observed for DTSQs scale score and its individual items at week 104, the primary time point in the study. However, significant treatment differences in favor of empagliflozin were observed at other endpoints, namely weeks 52 and 78 for DTSQs scale score and treatment recommendation, at week 52 for treatment flexibility, and at week 78 for current treatment satisfaction and treatment convenience (Table 3).

**Table 3:** Mean baseline and adjusted mean change from baseline in DTSQs scale score and its individual items

Scale score time point	Empagliflozin 25 mg^a^	Glimepiride 1–4 mg^a^	Treatment difference between empagliflozin 25 mg and glimepiride 1–4 mg^b^
DTSQs scale score			
Baseline [mean (SD)]	30.6 (5.5)	30.4 (5.4)	
Change from baseline^c^			
Week 8	1.3	1.3	0.0 (−0.4, 0.4) [*P* = 0.9741]
Week 28	1.6	1.5	0.1 (−0.3, 0.5) [*P* = 0.5070]
Week 52	2.2	1.7	0.5 (0.1, 0.9) [*P* = 0.0144]
Week 78	2.1	1.7	0.4 (0.0, 0.8) [*P* = 0.0398]
Week 104	2.3	2.1	0.2 (−0.2, 0.6) [*P* = 0.2991]
Current treatment satisfaction score			
Baseline [mean (SD)]	5.11 (1.16)	5.05 (1.16)	
Change from baseline^c^			
Week 8	0.28	0.25	0.03 (−0.05, 0.11) [*P* = 0.4840]
Week 28	0.36	0.29	0.07 (−0.01, 0.16) [*P* = 0.1037]
Week 52	0.39	0.32	0.08 (−0.01, 0.16) [*P* = 0.0823]
Week 78	0.43	0.33	0.10 (0.01, 0.18) [*P* = 0.0301]
Week 104	0.42	0.39	0.03 (−0.06, 0.12) [*P* = 0.5620]
Treatment convenience score			
Baseline [mean (SD)]	5.06 (1.20)	5.05 (1.14)	
Change from baseline^c^			
Week 8	0.23	0.23	−0.00 (−0.09, 0.09) [*P* = 0.9840]
Week 28	0.24	0.23	0.02 (−0.08, 0.11) [*P* = 0.7230]
Week 52	0.36	0.27	0.09 (−0.01, 0.18) [*P* = 0.0818]
Week 78	0.39	0.29	0.10 (0.00, 0.20) [*P* = 0.0468]
Week 104	0.38	0.33	0.05 (−0.05, 0.15) [*P* = 0.3159]
Treatment flexibility score			
Baseline [mean (SD)]	4.99 (1.27)	5.01 (1.22)	
Change from baseline^c^			
Week 8	0.15	0.25	−0.09 (−0.20, 0.01) [*P* = 0.0804]
Week 28	0.24	0.22	0.02 (−0.09, 0.13) [*P* = 0.7172]
Week 52	0.41	0.28	0.13 (0.02, 0.23) [*P* = 0.0224]
Week 78	0.29	0.25	0.04 (−0.07, 0.15) [*P* = 0.5156]
Week 104	0.39	0.34	0.05 (−0.06, 0.16) [*P* = 0.3578]
Satisfaction with understanding diabetes score			
Baseline [mean (SD)]	4.91 (1.19)	4.82 (1.16)	
Change from baseline^c^			
Week 8	0.27	0.23	0.04 (−0.04, 0.13) [*P* = 0.3180]
Week 28	0.32	0.29	0.03 (−0.06, 0.12) [*P* = 0.4947]
Week 52	0.43	0.36	0.07 (−0.02, 0.16) [*P* = 0.1211]
Week 78	0.44	0.39	0.05 (−0.04, 0.14) [*P* = 0.2995]
Week 104	0.47	0.47	0.01 (−0.09, 0.10) [*P* = 0.9114]
Treatment recommendation score			
Baseline [mean (SD)]	5.24 (1.19)	5.24 (1.17)	
Change from baseline^c^			
Week 8	0.18	0.16	0.03 (−0.06, 0.11) [*P* = 0.5436]
Week 28	0.24	0.24	0.00 (−0.08, 0.09) [*P* = 0.9577]
Week 52	0.34	0.25	0.09 (0.00, 0.18) [*P* = 0.0475]
Week 78	0.33	0.22	0.11 (0.03, 0.20) [*P* = 0.0113]
Week 104	0.35	0.27	0.07 (−0.02, 0.16) [*P* = 0.1088]
Treatment continuation score			
Baseline [mean (SD)]	5.30 (1.09)	5.26 (1.08)	
Change from baseline^c^			
Week 8	0.21	0.19	0.02 (−0.06, 0.10) [*P* = 0.6042]
Week 28	0.22	0.20	0.03 (−0.06, 0.11) [*P* = 0.5234]
Week 52	0.28	0.20	0.08 (−0.01, 0.16) [*P* = 0.0787]
Week 78	0.24	0.18	0.06 (−0.03, 0.14) [*P* = 0.1804]
Week 104	0.27	0.23	0.04 (−0.04, 0.13) [*P* = 0.3469]

*DTSQs* Diabetes Treatment Satisfaction Questionnaire, status version, *SD* standard deviation

^a^All within-treatment changes from baseline were significant (*P* < 0.001)

^b^Data presented in this column represent difference in adjusted mean changes from baseline, 95 % confidence interval, and *P* value

^c^Final adjusted model for change from baseline contained visit, treatment, treatment-by-visit interaction, baseline DTSQs item score, baseline diastolic blood pressure, and race as fixed effects and random intercept by subject

Table 4 presents results for perceived hyperglycemia and hypoglycemia. The final adjusted model for perceived hyperglycemia included baseline hyperglycemia score, age, country, diastolic blood pressure, and time since diagnosis. Significant decreases in perceived hyperglycemia were observed in each treatment group at all visits. Patients treated with empagliflozin showed more pronounced changes from baseline in perceived hyperglycemia than patients treated with glimepiride at all visits; the difference between the treatment groups was significant from week 28 onwards (Table 4). The final adjusted model for perceived hypoglycemia included baseline hypoglycemia score, age, body mass index, HbA1c, and diastolic and systolic blood pressure. Compared to baseline, a significant increase in perceived hypoglycemia was observed in the glimepiride treatment group at all visits, whereas patients treated with empagliflozin showed no significant increase from baseline in perceived hypoglycemia at any visit. The difference between the treatment groups was significant and in favor of empagliflozin from week 28 onwards (Table 4).

**Table 4:** Mean baseline and adjusted mean change from baseline in perceived hyperglycemia and hypoglycemia

Scale score time point	Empagliflozin 25 mg	Glimepiride 1–4 mg	Treatment difference between empagliflozin 25 mg and glimepiride 1–4 mg^a^
Perceived frequency of hyperglycemia			
Baseline [mean (SD)]	2.50 (1.96)	2.46 (1.92)	
Change from baseline^b^			
Week 8	−0.90***	−0.77***	−0.13 (−0.30, 0.04) [*P* = 0.1324]
Week 28	−0.97***	−0.72***	−0.24 (−0.41, −0.07) [*P* = 0.0056]
Week 52	−1.05***	−0.74***	−0.30 (−0.48, −0.13) [*P* = 0.0006]
Week 78	−1.08***	−0.71***	−0.37 (−0.55, −0.20) [*P* < 0.0001]
Week 104	−0.93***	−0.67***	−0.26 (−0.44, −0.08) [*P* = 0.0039]
Perceived frequency of hypoglycemia			
Baseline [mean (SD)]	0.76 (1.43)	0.85 (1.53)	
Change from baseline^c^			
Week 8	0.04	0.17**	−0.14 (−0.28, 0.01) [*P* = 0.0719]
Week 28	−0.12*	0.20***	−0.32 (−0.47, −0.17) [*P* < 0.0001]
Week 52	−0.01	0.18***	−0.20 (−0.35, −0.05) [*P* = 0.0109]
Week 78	−0.10	0.12*	−0.22 (−0.37, −0.06) [*P* = 0.0055]
Week 104	−0.02	0.21***	−0.23 (−0.39, −0.07) [*P* = 0.0043]

*DTSQs* Diabetes Treatment Satisfaction Questionnaire, status version, *SD* standard deviation

* *P* value <0.05; ** *P* value <0.01; *** *P* value <0.001

^a^Data presented in this column represent difference in adjusted mean changes from baseline, 95 % confidence interval, and *P* value

^b^Final adjusted model for change from baseline contained visit, treatment, treatment-by-visit interaction, baseline DTSQs item score, country, age, baseline diastolic blood pressure, and time since diagnosis of type 2 diabetes mellitus as fixed effects and random intercept by subject

^c^Final adjusted model for change from baseline contained visit, treatment, treatment-by-visit interaction, baseline DTSQs item score, age, baseline body mass index, baseline HbA1c (glycated hemoglobin), baseline diastolic blood pressure, and baseline systolic blood pressure as fixed effects and random intercept by subject

## Discussion

The objective of this study was to compare the treatment satisfaction, as measured by DTSQs scores, between patients taking empagliflozin 25 mg and glimepiride 1–4 mg as add-on therapy to current metformin treatment. Overall patient satisfaction at baseline was relatively high in both treatment groups. Nevertheless, patient satisfaction still increased significantly during the study within each treatment group. Despite a positive trend, the adjusted mean change from baseline in overall satisfaction was not significantly higher in the empagliflozin arm than in the glimepiride arm at the final visit. Similar results were observed for the individual items used to calculate the overall treatment satisfaction. Significant differences in changes in DTSQs scale score and some of its individual items, in favor of empagliflozin, were observed at weeks 52 and 78. However, given the potential inconsistency of findings throughout the observation period, the exploratory nature of this analysis, and the multiple comparisons being tested, the results should be viewed with caution.

Consistent with the analyses of the investigator-reported data described in Ridderståle et al. [11] are the significant treatment differences for perceived hyperglycemia and hypoglycemia, in favor of empagliflozin, at all visits after week 8. The difference in the perceived hyperglycemia scores was due to a more pronounced improvement (i.e., reduction) in perceived hyperglycemia for empagliflozin patients than for glimepiride patients. The significant treatment difference in the perceived hypoglycemia scores was due to an increase in perceived hypoglycemia for glimepiride patients, while no negative trend was reported by patients treated with empagliflozin.

Gelhorn et al. [16], using a conjoint analysis that assessed patient preferences, showed that the most important factors that determined patients’ preferences for oral medication were the likelihood of hypoglycemic events; weight change, especially for patients taking two or more medications; the likelihood of gastrointestinal side effects or nausea; and medication efficacy. Although empagliflozin patients showed significant improvements in perceived frequency of hyperglycemia and hypoglycemia when compared with glimepiride patients, the sustained weight loss, the reduced number of confirmed hypoglycemic events, and the reduction observed in HbA1c seen in the EMPA-REG H2H-SU study did not translate into significant benefit in DTSQs scale score; therefore, further investigation is required.

Our study has several limitations. The DTSQs was used in a double-blind, double-dummy trial, and this design feature may mask the effect of treatment on individual items (i.e., treatment convenience, treatment flexibility, and satisfaction with understanding diabetes) and consequently may dilute potential effects on the scale score. Also, a ceiling effect was observed in this population with the DTSQs (i.e., patients who were already very satisfied at baseline on some or all items of the DTSQs have little or no room for improvement). There is a change version of the DTSQ, the DTSQc, which may overcome such ceiling effects [17, 18], but this was not used in the present study. To determine the clinical significance of a PRO measure, researchers try to derive the minimal clinically important difference. However, to our knowledge, no minimally important threshold has been established for the DTSQs. In a response written to the United States Food and Drug Administration, Bradley [19] stated that “a statistically significant difference on measures of treatment satisfaction that have been designed explicitly to measure issues of importance to patients (e.g., DTSQ) will necessarily be an important difference.”

In conclusion, although patients with T2DM treated with empagliflozin did not show a significant improvement in DTSQs scale score at 104 weeks compared with patients treated with glimepiride, a significant benefit in favor of empagliflozin with regard to perceived hyperglycemia and perceived hypoglycemia was observed at all visits from week 28 onward up to the final assessment. This finding is consistent with the clinical results reported for the EMPA-REG H2H-SU trial.
